# Comparative transcriptome analysis of high- and low-embryogenic *Hevea brasiliensis* genotypes reveals involvement of phytohormones in somatic embryogenesis

**DOI:** 10.1186/s12870-023-04432-3

**Published:** 2023-10-13

**Authors:** Ling Li, Xiaolong Sun, Wencai Yu, Mingchun Gui, Yanfen Qiu, Min Tang, Hai Tian, Guoping Liang

**Affiliations:** The Center of Rubber Research, Yunnan Institute of Tropical Crops, Xishuangbanna, China

**Keywords:** Abscisic acid, Auxins, Cytokinins, Ethylene, H_2_O_2_, MAPK signaling, Somatic embryogenesis

## Abstract

**Background:**

Rubber plant (*Hevea brasiliensis*) is one of the major sources of latex. Somatic embryogenesis (SE) is a promising alterative to its propagation by grafting and seed. Phytohormones have been shown to influence SE in different plant species. However, limited knowledge is available on the role of phytohormones in SE in Hevea. The anther cultures of two Hevea genotypes (Yunyan 73477-YT and Reken 628-RT) with contrasting SE rate were established and four stages i.e., anthers (h), anther induced callus (y), callus differentiation state (f), and somatic embryos (p) were studied. UPLC-ESI-MS/MS and transcriptome analyses were used to study phytohormone accumulation and related expression changes in biosynthesis and signaling genes.

**Results:**

YT showed higher callus induction rate than RT. Of the two genotypes, only YT exhibited successful SE. Auxins, cytokinins (CKs), abscisic acid (ABA), jasmonic acid (JA), salicylic acid (SA), gibberellins (GAs), and ethylene (ETH) were detected in the two genotypes. Indole-3-acetic acid (IAA), CKs, ABA, and ETH had notable differences in the studied stages of the two genotypes. The differentially expressed genes identified in treatment comparisons were majorly enriched in MAPK and phytohormone signaling, biosynthesis of secondary metabolites, and metabolic pathways. The expression changes in IAA, CK, ABA, and ETH biosynthesis and signaling genes confirmed the differential accumulation of respective phytohormones in the two genotypes.

**Conclusion:**

These results suggest potential roles of phytohormones in SE in Hevea*.*

**Supplementary Information:**

The online version contains supplementary material available at 10.1186/s12870-023-04432-3.

## Background

The Rubber tree (*Hevea brasiliensis* Muell. Arg. hereafter Hevea) is a tropical tree that was first discovered in the Amazonian basin of South America [1]. It is a member of the *Euphorbiaceae* family. It is frequently farmed in Southeast Asia to produce natural rubber. One of the major sources of latex is the Hevea, which serves as the basis for more than 40,000 products [2]. Hevea multiplication has been hampered by its lengthy growth cycles, sever inbreeding depression upon self-pollination, cross-pollination, recalcitrant seed, and poor seed germination [3, 4]. As a result, the tree is mostly propagated through grafting. However, the growth and natural rubber yield of a grafted tree can be influenced by the interaction between its scion and rootstock [4]. Somatic embryogenesis (SE) is a quick and efficient technique for plant regeneration and propagation [5]. SE has been successfully achieved in the case of rubber trees by employing a variety of explants including roots, inflorescences, internal integuments of immature fruits, and anthers [6, 7]. Self-rooted juvenile clones (SRJCs) are plants that have grown through the SE process and have their own roots and juvenile characteristics. In comparison to donor clones (DCs), SRJCs exhibit higher stress resistance, growth, and rubber yield, making them a promising option for future rubber tree planting material [5, 7, 8]. Considering urgent need for the development of natural rubber industry, it essential to develop somatic embryos propagated plants for the excellent varieties of rubber trees. However, rubber plant regeneration through SE is largely dependent on the genotype [9–11]. Consequently, the ability to obtain regenerated plants has been restricted to a narrow range of rubber tree genotypes [5, 12–15].

Phytohormones play a crucial role in somatic cell embryogenesis in plants. Auxins, cytokinins (CKs), and abscisic acid (ABA) are the most critical hormones involved in this process [16]. Several investigations have found that auxin and CK ratios are critical for determining the developmental fate of somatic cells during embryogenesis [17]. High levels of auxin and low levels of CK are required for the induction of SE, while a balance of both hormones is needed for the development of somatic embryos [18]. ABA has also been implicated in the regulation of embryo growth and maturation in SE [19]. Other hormones such as gibberellins (GAs) and ethylene (ETH) may also influence SE, but their roles are not yet fully understood. Overall, the complex interactions of various hormones are essential for the successful induction and development of somatic embryos in plants.

The ability to achieve SE in rubber tree is limited to certain genotypes, as documented in earlier studies [12–14]. Despite the significance of SE in rubber tree, there is limited understanding of the molecular regulatory mechanism driving this biological process. A pioneering study (two decade ago) identified five differentially expressed cDNAs in an embryogenically regenerating Hevea line that could potentially aid in early diagnosis of the embryogenic potential of friable rubber tree callus [12]. However, the functions of these cDNAs remain unknown. In another study [20], researchers used transcriptome analysis to investigate the molecular mechanisms underlying SE in Hevea. However, the analysis was limited to a single genotype. To investigate the molecular regulatory mechanisms of plant SE, RNA-seq analyses have been performed to identify SE-related genes in various plant species, including Arabidopsis [21], *Gossypium hirsutum* [22], maize [23], strawberry [24], rice [25], coconut palm [26], and Norway spruce [27]. These studies revealed regulatory mechanisms of SE at the molecular level and identified potential key genes such as somatic embryogenesis receptor-like kinase (SERK) [28], late embryogenesis abundant (LEA) protein [29], AGAMOUS-like 15 (AGL15), leafy cotyledon, WUSCHEL (WUS), WUS-homeobox 2, and Baby Boom(BBM) ([30] and references therein, [31]). Li*, *et al*.* [32] identified three differentially expressed MADS-box genes during early embryogenesis in rubber tree. Another study reported 11 AP2/ERF genes that may serve as expression markers for different stages of the SE process in rubber tree [14]. Other studies have also reported that AP2/ERF genes are involved in regulation of SE [33–35]. However, the molecular mechanisms regulating SE and the effects of phytohormones on genotypes with different SE capabilities remain poorly understood.

In this study, we explored the endogenous phytohormone content of two *H. brasiliensis* genotypes differing in SE potential. We used transcriptome sequencing analysis to explore the expression differences in the two genotypes regarding phytohormone biosynthesis and signaling.

## Methods

### Plant material

Two *H. brasiliensis* varieties, Yunyan 73477 (YT) and Reken 628 (RT), were used as plant material. The varieties YT and RT exhibit high and low SE, respectively. However, the latter is the main variety in rubber planting areas in China, therefore, it is an important resource to be improved. The plant materials were obtained from the Genebank of Yunnan Institute of Tropical Crops. They are conserved at the Genebank of Yunnan Institute of Tropical Crops under voucher numbers YITC4002 and YITC4009. No permission is needed to study these accessions. The formal identification of plant materials was undertaken by Prof Guoping Liang. Male flowers were used as starting material. Male flowers were soaked in 75% (v/v) ethanol for 30 s, then soaked in 0.1% HgCl_2_ (w/v) for 10 min followed by five rinses with distilled water. The YT immature anthers were isolated and inoculated on solid callus induction medium (MS medium containing 2,4-D 1 mg L^−1^, KT 1.0 mg L^−1^, NAA 1.0 mg L^−1^, sucrose 70 g L^−1^ and coconut water 50 ml L^−1^) for 50 days. Then transferred to a differentiation medium (solid MS medium with activated charcoal 1.0 g L^−1^, KT 2.0 mg L^−1^, NAA 0.1 mg L^−1^, GA_3_ 0.5 mg L^−1^, ABA 0.2 mg L^−1^, sucrose 70 g L^−1^ and coconut water 50 ml L^−1^) to induce somatic embryos. After 25 days of differentiation, embryonic calli with spherical embryos were obtained. After differentiation and cultivation for 60 days, somatic embryos in cotyledonary stage were obtained. The RT anthers were inoculated for 50 days to induce calli and the non-embryonic calli were cultured for 25 days. Callus induction rate and SE rate were measured according to the following equations.

$$Callus\ induction\ rate=\frac{No.\ of\ anther\ explants\ forming\ callus}{number\ of\ anther\ explants\ inoculated}\times 100\%$$

$$Somatic\ embryogenesis\ rate=\frac{No.\ of\ calli\ with\ embryogenesis\ ability}{number\ of\ calli\ inoculated}\times 100\%$$

Triplicate samples of male flower (RT-h), and two callus stages: anther callus induction stage (RT-y) and differentiation culture stage (RT-f), were taken for RT, frozen in liquid nitrogen, and stored at -80 ℃ until further processing. Similarly, triplicate samples of male flower (YT-h, two callus stages: anther callus induction stage (YT-y) and differentiation culture stage (YT-f), and somatic embryo (YT-p) were collected for YT, frozen in liquid nitrogen, and stored at -80 ℃ until further processing.

### Endogenous plant hormone analysis

Reagents were purchased from Merck (Darmstadt, Germany), Millipore (Bradford, USA), Olchemim Ltd. (Olomouc, Czech Republic), isoReag (Shanghai, China), and Sigma-Aldrich (St Louis, MO, USA). Stock solutions of the standards were prepared at a concentration of 1 mg/mL in methanol (MeOH) and stored at -20 ℃. Working solutions were prepared from the stock solutions by diluting with MeOH.

From samples stored at -80 ℃, 50 mg of each sample (each replicate) was ground to powder (30 Hz, 1 min), followed by extraction in 1 mL MeOH/water/formic acid (15:4:1, V/V/V). For quantification, we added 10 μL of internal standard mixed solution (100 ng/mL) to the extracts as internal standards (IS). The mixture was vortexed for 10 min, centrifuged at 12,000 r/min at low temperature (4 ℃) for 5 min, and the supernatants were transferred to clean plastic microtubes. The supernatants were dried by evaporation, dissolved in 100 μL 80% MeOH (v/v), and filtered through a 0.22 μm Durapor ® membrane filter (Merck KGaA, Darmstadt, Germany). The filtrates were then used for LC–MS/MS analyses using an UPLC-ESI–MS/MS system (UPLC, ExionLC™ AD,; MS, Applied Biosystems 6500 Triple Quadrupole, https://sciex.com.cn/). The analytical conditions were set as follows, LC: column, Waters ACQUITY UPLC HSS T3 C18 (100 mm × 2.1 mm, 1.8 µm); the solvent system included water with 0.04% acetic acid (A) and acetonitrile with 0.04% acetic acid (B); the gradient program started at 5% B for 0–1 min, increased to 95% B for 1–8 min, maintained at 95% B for 8–9 min, and finally decreased to 5% B for 9.1–12 min; the flow rate was 0.35 mL/min; the temperature was 40 °C, and the injection volume was 2 μL. Linear ion trap and triple quadrupole scans were acquired on a triple quadrupole-linear ion trap (QTRAP) mass spectrometer, QTRAP® 6500 + LC–MS/MS system. The system was equipped with an ESI Turbo Ion-Spray interface, operated in two ion modes: positive and negative, and controlled by Analyst 1.6.3 software (Sciex). The operating parameters of the ESI source were as reported earlier [36]. Phytohormones were analysed by scheduled multiple reaction monitoring (MRM) and data acquisitions were performed using Analyst 1.6.3 software. Multiquant 3.0.3 software (Sciex) was used for quantification. All the mass spectrometer parameters including the declustering potentials (DP) and collision energies (CE) for individual MRM transitions were performed with further DP and CE optimisation. A specific set of MRM transitions was monitored for each time period according to the metabolites eluted within that time period.

### Statistical and bioinformatic analysis of phytohormone data

Hierarchical cluster analysis (HCA) was performed using the R package pheatmap and presented as heat maps of normalised signal intensities of metabolites with dendrograms. Significantly differentially accumulated metabolites (DAMs) between groups were determined by absolute Log2FC (fold change). The DAMs were annotated using the KEGG compound database (http://www.kegg.jp/kegg/compound/) and mapped to the KEGG pathway database (http://www.kegg.jp/kegg/pathway.html). Pathways with significantly regulated metabolites mapped were then fed into MSEA (metabolite sets enrichment analysis), their significance was determined by hypergeometric test’s *p*-values.

### Transcriptome sequencing

#### RNA extraction, library preparation, and sequencing

Total RNAs were extracted from the triplicate samples of each tissue of the two genotypes using a Spin Column lant total RNA Purification Kit (Tiandz, Beijing, China) [37]. The RNA purity and integrity were checked, and sequencing libraries were prepared as previously reported [36]. The libraries were sequenced on the Illumina sequencer.

#### Sequencing data analysis

The quality of the sequencing libraries was checked using fastp [38] and removed reads with adapters, if the N content in the sequencing read exceeded 10% of the number of bases in the read, or if the number of low-quality bases (Q ≤ 20) in a sequencing read exceeded 50% of the number of bases in that read. This step was followed by checking the sequencing error rate distribution check and the GC content distribution. We used HISAT2 [39] to compare the clean reads to the reference genome [40]. Gene expression was calculated as Fragments Per Kilobase of transcript per Million fragments mapped (FPKM) and visualized as a boxplot. Pearson’s correlation coefficient (PCC) and principal component analysis (PCA) were calculated in R (www.rproject.com). Differential gene expression was calculated in DESeq2 [41] followed by correction of the hypothesis testing probability using the Benjamini–Hochberg method [42] for multiple hypothesis testing and to obtain the false discovery rate (FDR). The differential genes were screened using the criteria |log 2-fold change|> = 1, and FDR < 0.05. Heat maps and Venn diagrams were generated in tBtools [43]. Differentially expressed genes (DEGs) were annotated in the KEGG [44] and GO [45] databases. The DEGs were enriched in KEGG pathways [46, 47].

### Antioxidant enzyme activity and H_2_O_2_ content determination

The activities of the antioxidant enzymes, peroxidase (POD), superoxide dismutase (SOD), and catalase (CAT), and the content of H_2_O_2_ in the immature anther culture stages of YT and RT were studied according to established methods [48]. Whereas, the ascorbate peroxidase (APX) activity was determined according to the method used in an earlier study [49].

## Results

### Genotype dependent SE in *H. brasiliensis*

**Fig. 1:**
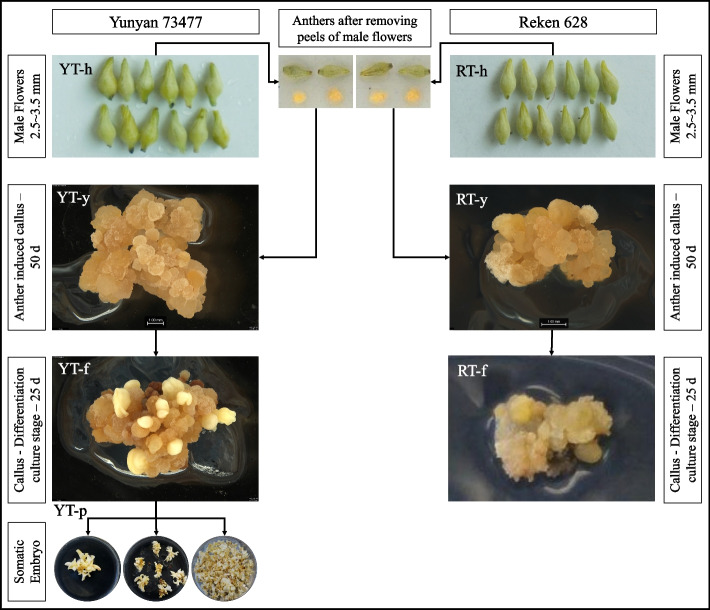
Immature anther culture of two *H. brasiliensis* varieties: Yunyan 73477 (YT) and Reken 628 (RT) differing in SE ability. SE: somatic embryogenesis, h: immature anther, y: anther induced callus, f: callus differentiation stage, p: cotyledonary embryo

**Fig. 2:**
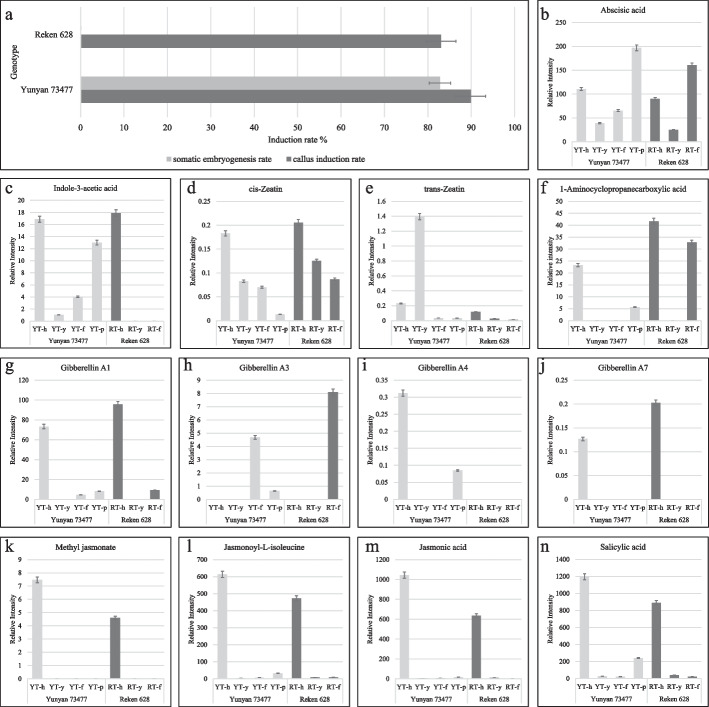
**a** Callus induction rate and SE rate in Yunyan 73477 (YT) and Reken 628 (RT). **b**-**n** Relative metabolite intensities of phytohormones in anthers (**h**), anther induced callus (**y**), callus differentiation state (**f**), and somatic embryos (**p**) in YT and RT

The process of SE in two *H. brasiliensis* genotypes i.e., YT and RT was established following a previously described method [50] (Fig. 1). Both varieties of anthers (h) induced callus (y) in MS medium containing 2,4D, KT, and NAA. The callus induction rate of genotype YT (89.9%) was higher than that of genotype RT (82.8%). Twenty-five days after the callus was transferred to the differentiation medium containing GA3, ABA, high KT content and low NAA content (that is, somatic cell embryo induction culture), it was observed that only YT callus had SE ability. Its embryogenic callus had spherical or cardioid cell embryos (YT-f), and the somatic embryo induction rate was 83.04%. After 60 days of differentiation and culture, cotyledon type SEs (YT-p) were obtained. But RT callus was not embryogenic (RT-f). After differentiation and culture, no somatic cell embryogenesis occurred (Fig. 2a).

### UPLC-ESI–MS/MS based phytohormone detection in the two genotypes

The employed system detected seven major phytohormones: auxins, CKs, ABA, jasmonic acid (JA), salicylic acid (SA), GAs, and ETH and their conjugates/precursors (Supplementary Table S1). We observed that the relative content of ABA was lower in YT-y and YT-f compared to YT-h, while in the case of YT-p, the content was 43.78% higher than YT-h. In the case of RT, the relative ABA content was lower in RT-y compared to RT-h, however, it was higher in RT-f. Overall, we observed that the relative ABA content was higher in YT-h and YT-y compared to the RT-h and RT-y. However, in the case of differentiated callus (f), the ABA content was higher in RT than YT.

During SE, the levels of ABA glycosyl ester varied (Supplementary Table S1). The pattern of changes observed in ABA glycosyl ester levels was similar to that of ABA, except for in the case of YT-p, where ABA glycosyl ester levels were lower than ABA. These observations suggest potential conversion of ABA glycosyl ester into ABA during SE. The levels of IAA differed in YT-h and RT-h such that the latter had slightly higher content than the former. Interestingly, the IAA content decreased from YT-h to YT-y and then increased in YT-f and YT-p. However, despite the increase in latter stages, the content remained lower than in YT-h. On the contrary, we did not detect IAA in RT-y neither in RT-f. This is an important observation and may be a possible reason for the different SE capacity of the two genotypes.

There were 19 metabolites related to auxin (Supplementary Table S1, Supplementary Figure S1). Among them, L-tryptophan was highest in YT-h and RT-h stages (YT-h with higher content). The relative content of indole-3-acetyl-L-aspartic acid was highest in YT-f and YT-p. However, 1-O-indol-3-ylacetylglucose was the major IAA-component in YT-h, RT-h, and RT-y. For CKs 28 metabolites were identified. These were classified into precursors, storage forms, byproducts, degradation products, and intermediates (Supplementary Figure S2). Two major CKs i.e., cis-Zeatin (cZ) and trans-Zeatin (tZ) showed different accumulation trends in the two genotypes. We observed that the relative content of cZ decreased in both genotypes with the progression of the callus induction, redifferentiation, and SE (in YT), whereas, tZ relative content showed a similar trend in RT but not in YT (Fig. 2). The tZ content in RT-h was relatively lower than YT-h. In case of RT, tZ content decreased in the following stages whereas in case of YT, its content significantly increased from YT-h to YT-y. However, in the latter stages i.e., YT-f and YT-p, it decreased compared to YT-h. In YT, the precursor of CK (4-[[(9-beta-D-Glucopyranosyl-9H-purin-6-l) amino]methyl]phenol (also known as isopentenyladenine-adenine) followed an increasing trend from YT-h to YT-f and then decreased in YT-p. However, in the case of RT, it was detected from RT-y and RT-f (Supplementary Table S1). Various forms of active CK (kinetin, dihydrozeatin, cZ riboside, tZ riboside, meta-topolin riboside, 2-methylthio-cZ riboside, and dihydrozeatin ribonucleoside) were detected in different SE stages.

The relative content of ETH showed variable trends in both genotypes. In YT, it was detected only in “h” and “p” stages, whereas in case of RT, it was detected in “h” and “f” stages. The absence of ETH in YT-f and its high content in RT-f may suggest that it is negatively related to SE. Apart from these hormones, we also detected ten GAs including GA1, 3, 4, 7, 9, 15, 19, 20, 24, and 53 (Supplementary Table S1). Among the active GAs, GA1 and 7 had higher contents in YT-h and RT-h but were almost absent in other stages. The GA1 was also detected in YT-f and RT-f suggesting limited roles in SE. In the case of GA3, YT-f and RT-f showed accumulation but not in other stages. GA4 was detected only in YT-h and YT-p. These observations suggest that GAs have a limited role in the SE of Hevea.

Finally, we detected the differential accumulation of nine metabolites classified as JA or conjugates (Supplementary Table S1). Of these, we observed that YT-h and RT-h had higher levels of the major Jas: methyl jasmonate (MeJA), jasmonoyl-isoleucine (JA-Ile), and JA. The absence or detection of negligible amounts of JA and JA-Ile indicates limited role of JA in SE.

### Transcriptome analysis of *H. brasiliensis* during SE

**Fig. 3:**
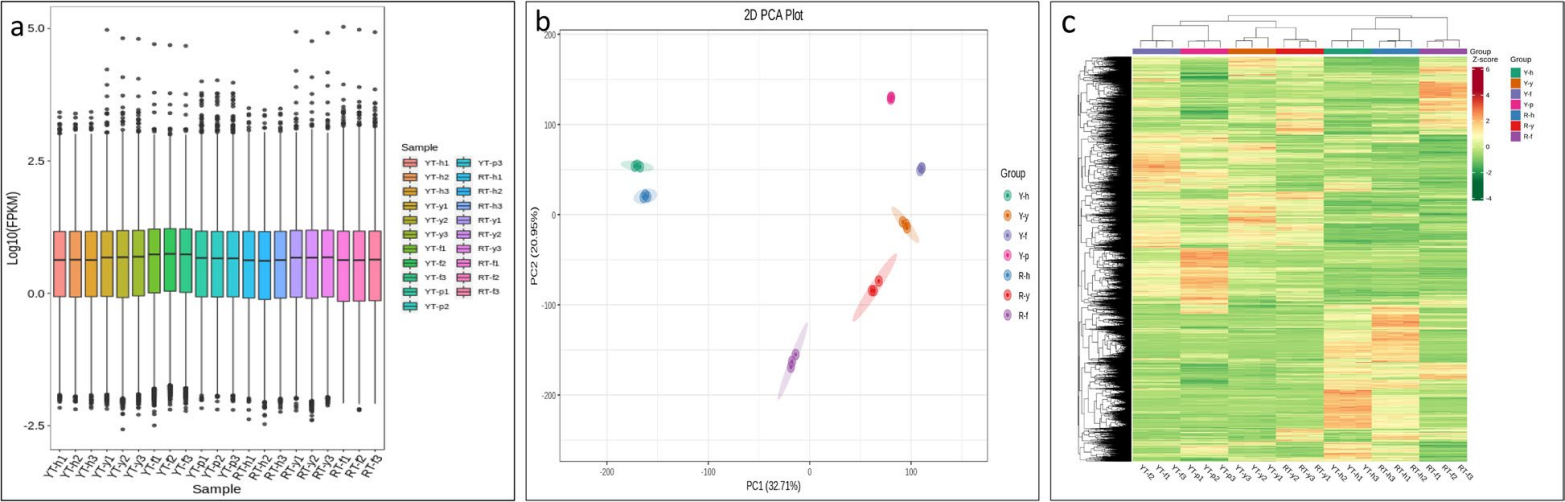
Transcriptome statistics. **a** FPKM distribution boxplot of each sample in this project. **b** Principal component analysis. **c** Heat map of differentially expressed transcripts. SE: Somatic embryogenesis, h: Immature Anther, y: Anther-induced callus, f: Callus differentiation stage, p: Cotyledonary embryo; YT: genotype- Y or Yunyan 73477; RT: RT or Reken 628

The RNA purity and integrity were evaluated, and overall high-quality RNA samples were used for transcriptome sequencing (Supplementary Table S2). The transcriptome sequencing analysis of 21 samples was completed, and a total of 186.64 Gb of clean data was obtained with an average of 7 Gb clean data per sample. A total of 1,244,105,756 clean reads were obtained after quality control. The percentage of Q30 bases was > 93% and the percentage of GC was 43% (Supplementary Table S2). Of the clean reads ~ 94% could be mapped to the *H. brasiliensis* genome*.* A total of 39,894 unigenes were identified and annotated using KEGG, NR, Swissprot, Trembl, KOG, GO, and Pfam databases. The overall gene expression levels of the different samples are shown as a box plot (Fig. 3A). PCA showed that all the replicates for each sample were close to each other. The PC1 and PC2 represented 20.95% and 32.71% variability, respectively (Fig. 3b). A heat map was generated to evaluate the uniformity of replicates for gene expression, and it was observed that the replicates were clustered together (Fig. 3c).

### Differential gene expression in *H. brasiliensis* genotypes YT and RT during SE

**Fig. 4:**
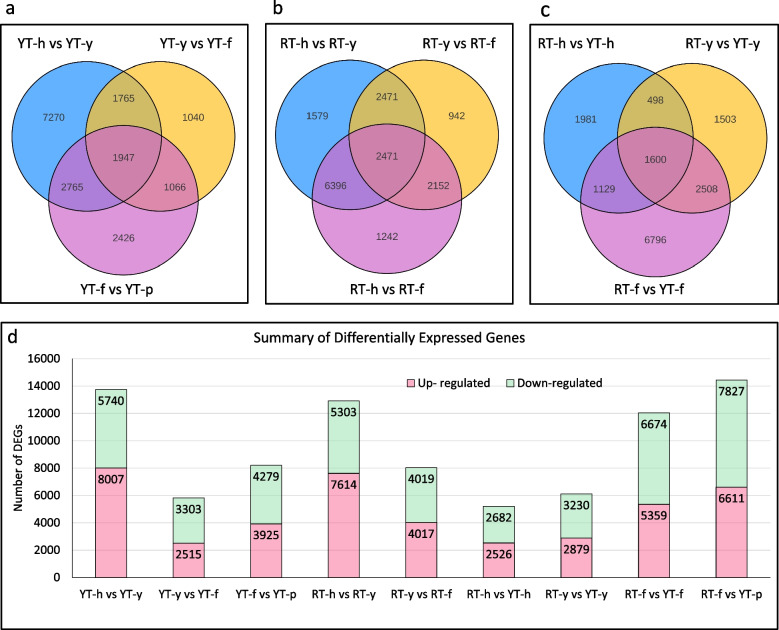
Summary of the DEGs during SE stages. **a** The Venn diagram of DEGs in SE stages in YT, **b** RT, **c** between both genotypes at relevant SE stage, **d** Number of differentially up-regulated and down-regulated genes in different stages of SE in both genotypes. SE: Somatic embryogenesis, h: Immature Anther, y: Anther induced callus, f: Callus differentiation stage, p: Cotyledonary embryo; Y: genotype- Y or Yunyan 73477; R: genotype-R or Reken 628

A Venn diagram was used to illustrate overlapping or distinctly expressed genes at h, y, f and p stages of SE in both genotypes (Fig. 4). The top 10 most expressed genes in RT-h, RT-y, RT-f, YT-h, YT-y, YT-f, and YT-p are enlisted in Table 1. The *Extensin-3-like* gene was among the top 10 most expressed genes in all the samples. Therefore, it can be a good candidate for marker studies. Similarly, *SRC1-like* (*LOC110638422*) was uniformly expressed in all samples except for RT-f. The top-5 most expressed genes at YT-p (*LOC110647147, LOC110659495, LOC110662110, LOC110635440*) had very low or no expression in all stages, suggesting their essential role in the formation of cotyledonary embryos.

**Table 1:** List of top 10 highly expressed genes in the studied stages of YT and RT genotypes

	**ID**	**FPKM**	**Description**
**YT-h**	*LOC110636006*	2588.678	Extensin-3-like
	*LOC110633183*	2086.292	hypothetical protein PHAVU_003G123500g
	*LOC110635649*	1589.129	heat shock cognate 70 kDa protein 2-like isoform X1
	*LOC110638422*	1560.971	protein SRC1-like
	*LOC110670171*	1408.031	non-specific lipid-transfer protein 1-like
	*LOC110665310*	1293.447	glucan endo-1,3-beta-glucosidase, basic isoform-like
	*LOC110644721*	1275.933	S-adenosylmethionine synthase 1
	*LOC110641104*	1219.316	blue copper protein-like
	*LOC110639317*	1025.061	uncharacterized protein LOC110639317
	*LOC110669847*	1020.857	probable calcium-binding protein CML27
**YT-y**	*LOC110659535*	74,560.68	repetitive proline-rich cell wall protein 2-like
	*LOC110661350*	13,392.07	early nodulin-75-like
	*LOC110636006*	6438.401	extensin-3-like
	*LOC110654674*	3805.296	36.4 kDa proline-rich protein-like isoform X2
	*LOC110664540*	2513.964	putative lipid-transfer protein DIR1
	*LOC110631692*	2396.46	probable indole-3-acetic acid-amido synthetase GH3.1
	*LOC110638422*	2317.033	protein SRC1-like
	*LOC110663547*	2311.606	early nodulin-75-like
	*LOC110661355*	1963.759	early nodulin-75-like
	*LOC110658306*	1744.359	alcohol dehydrogenase 1-like
**YT-f**	*LOC110659535*	48,787.11	repetitive proline-rich cell wall protein 2-like
	*LOC110662584*	5987.843	metallothionein-like protein type 2
	*LOC110636006*	3620.276	extensin-3-like
	*LOC110638422*	2498.915	protein SRC1-like
	*LOC110658306*	1279.519	alcohol dehydrogenase 1-like
	*LOC110641169*	1262.098	extensin-2-like
	*LOC110634872*	1222.233	polyubiquitin 10
	*LOC110647147*	1112.778	2S albumin
	*LOC110670171*	1000.166	non-specific lipid-transfer protein 1-like
	*LOC110632873*	960.3227	auxin-repressed 12.5 kDa protein-like
**YT-p**	*LOC110647147*	10,037.63	2S albumin
	*LOC110659495*	6039.969	legumin B-like
	*LOC110662110*	5795.481	legumin A-like
	*LOC110635440*	5286.086	legumin B-like
	*LOC110638422*	4604.952	protein SRC1-like
	*LOC110636006*	4409.602	extensin-3-like
	*LOC110658834*	3918.858	metallothionein-like protein type 2
	*LOC110670171*	3514.715	non-specific lipid-transfer protein 1-like
	*LOC110644520*	2257.456	uncharacterized protein LOC110644520
	*LOC110647335*	2118.786	pathogenesis-related protein PR-4-like isoform X1
**RT-h**	*LOC110636006*	3041.87	extensin-3-like
	*LOC110640545*	2380.113	(S)-hydroxynitrile lyase
	*LOC110633183*	2314.983	hypothetical protein PHAVU_003G123500g
	*LOC110670171*	1689.63	non-specific lipid-transfer protein 1-like
	*LOC110660159*	1442.091	metallothionein-like protein type 2
	*LOC110635649*	1351.951	heat shock cognate 70 kDa protein 2-like isoform X1
	*LOC110638422*	1243.194	protein SRC1-like
	*LOC110662584*	1124.023	metallothionein-like protein type 2
	*LOC110639369*	1116.595	beta-glucosidase 24-like
	*LOC110634872*	1052.264	polyubiquitin 10
**RT-y**	*LOC110659535*	75,848.19	repetitive proline-rich cell wall protein 2-like
	*LOC110661350*	14,242.68	early nodulin-75-like
	*LOC110660159*	7324.757	metallothionein-like protein type 2
	*LOC110636006*	3986.75	extensin-3-like
	*LOC110631692*	2508.395	probable indole-3-acetic acid-amido synthetase GH3.1
	*LOC110672503*	1884.62	cytochrome P450 71A1-like
	*LOC110638422*	1694.775	protein SRC1-like
	*LOC110652862*	1597.531	L-ascorbate peroxidase, cytosolic-like isoform X1
	*LOC110643427*	1493.771	indole-3-acetic acid-amido synthetase GH3.3-like
	*LOC110646012*	1476.988	phosphoenolpyruvate carboxykinase (ATP)-like
**RT-f**	*LOC110659535*	96,003.64	repetitive proline-rich cell wall protein 2-like
	*LOC110660159*	7908.655	metallothionein-like protein type 2
	*LOC110647926*	4163.812	thaumatin-like protein 1b
	*LOC110631692*	3333.439	probable indole-3-acetic acid-amido synthetase GH3.1
	*LOC110636006*	3241.049	extensin-3-like
	*LOC110655007*	2694.334	glutathione S-transferase L3-like isoform X2
	*LOC110647329*	2618.172	pathogenesis-related protein PR-4-like
	*LOC110661350*	2548.735	early nodulin-75-like
	*LOC110662584*	2347.88	metallothionein-like protein type 2
	*LOC110655226*	2268.535	thioredoxin H-type-like

In order to reveal the potential key factors and deeply understand the regulatory network of SE, the DEGs for each stage were identified (Fig. 4); only 1,947 and 2,471 genes were commonly expressed among all these stages in genotypes YT and RT, respectively. These might be the distinct set of genes in respective genotypes. A total of 5,208, 834, 762, 1,705, and 434 unique DEGs were identified for RT-h vs YT-h, RT-y vs YT-y, R-f vs YT-f, RT-f vs YT-p and YT-f vs YT-p, respectively. It is important to note that significant phenotypic variations (for embryogenesis) appear between both genotypes at primary embryo (y stage, Fig. 1). In YT, 2515 (up-regulated) and 3,303 (down-regulated) DEGs were identified for YT-y vs YT-f (Fig. 4d). Of these, 1,040 were exclusively expressed during YT-y vs YT-f (Fig. 4a). Similarly, 3,925 and 4,279 DEGs were respectively up- and down-regulated in YT-f vs YT-p. In contrast, a relatively higher number of DEGs (4,017 up- and 4,019 down-regulated) were identified in RT-y vs RT-f. Only 942 of these DEGs were uniquely expressed at this stage (Fig. 4b).

For further interpretation of gene function, KEGG pathway annotation analysis of DEGs was performed. The DEGs were assigned to 138 and 136 KEGG pathways in RT-h vs RT-y, and RT-y vs RT-f, respectively (Supplementary Figures S3 and S4). The most representative pathways include metabolic pathways (1890 unigenes), biosynthesis of secondary metabolites (1202 unigenes), plant hormones signal transduction (506 unigenes), and MAPK signaling pathway-plant (378 unigenes). On the other hand, the DEGs in YT-h vs YT-y, YT-y vs YT-f, and YT-f vs YT-p were assigned to > 130 KEGG pathways (Supplementary Figure S4), which were mostly the same as described above. Interestingly, the DEGs identified in the treatment comparisons between the two genotypes i.e., RT-h vs YT-h, RT-y vs YT-y, RT-f vs YT-f, RT-f vs YT-p, were also common to the individual list of KEGG pathways for each genotype. However, the representative number of genes/transcripts was different for each comparison.

### Transcriptome analysis confirm the phytohormone accumulation trends

**Fig. 5:**
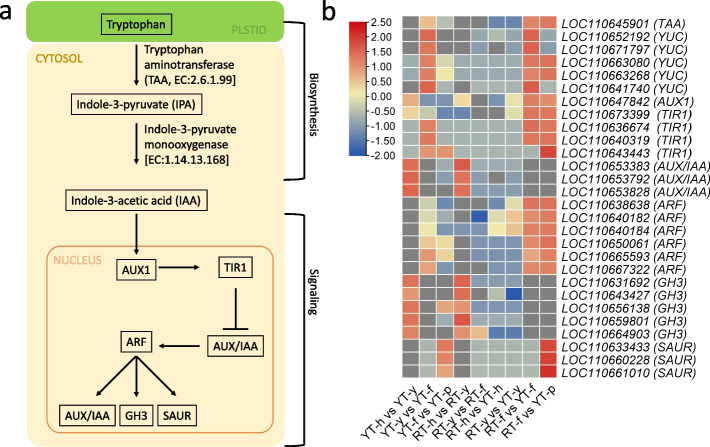
Auxin biosynthesis and signaling pathway. **a** representation of auxin biosynthesis and signaling pathway genes and metabolites. **b** Heatmap representation of DEGs enriched in auxin biosynthesis and signaling pathway. Red: up-accumulation, Blue: down-accumulation, yellow: no variation of expression. SE: Somatic embryogenesis, h: immature anther, y: anther induced callus, f: callus differentiation stage, p: cotyledonary embryo

To further elucidate expression changes related to hormone biosynthesis and signaling regulation, we explored the differential expression of related genes (Supplementary Table S3). Tryptophan aminotransferase (TAA) and YUCCA (YUC) enzymes (like indole-3-pyruvate monooxygenase [EC:1.14.13.168]) are the key genes involved in the auxin biosynthesis pathway. These genes showed relatively higher expression in YT as compared to RT in all four stages of SE (Fig. 5). From anther to callus induction, the differential expression of TAAs and YUCs was lower. However, compared to YT-h and YT-y, a significant increase in their expressions was observed in YT-f. This is the stage when the cells start to divide and undergo proliferation and differentiation. Moreover, the expression of these genes was higher in YT-f as compared to RT-f (Fig. 5). These changes in expression are consistent with the observed IAA accumulation in both genotypes.

We further looked at the expression changes in the auxin signaling pathway (as per KEGG pathway map ko04075) and found eight auxin transporter-like (*AUX1-like*) proteins, two of which (*LOC110647842, LOC110673399*) were highly up-regulated in YT during callus induction (Fig. 5). On the other hand, *LOC110633296, LOC110653578*, and *LOC110665570* were highly expressed in RT-f compared to YT-f. In addition, we observed upregulation of transport inhibitor response 1 (TIR1, *LOC110673399, LOC110636674, LOC110640319, and LOC110643443*) in YT-f and downregulation in RT-h. There were 34 DEGs annotated as AUX/IAA family proteins that showed variable expression trends. Generally, these transcripts were down-regulated during the “immature anther or h” stage, up-regulated during callus induction (YT-y and RT-y), and down-regulated again in later stages (Supplementary Table S3, Fig. 5).

Among 43 ARF DEGs, *LOC110638638, LOC110640182, LOC110640184, LOC110650061, LOC110665593, LOC110667322* were down-regulated in transition from YT-h to YT-y and then up-regulated in next stage. Interestingly, these transcripts were mostly down-regulated in RT. In general, the FPKM values of these genes were higher in YT compared to RT. Apart from the ARFs, we also observed the expression changes in GH3 and SAUR family genes. The GH3s (*LOC110631692, LOC110643427, LOC110656138, LOC110659801, LOC110664903*) were up-regulated during YT-h vs YT-y and down-regulated in later stages. A similar trend was observed for in RT-h vs RT-y and the later stages. However, between both genotypes, these genes were down-regulated in YT compared to RT. Moreover, we identified 46 DEGs annotated as SAURs, of which *LOC110633433, LOC110660228, LOC110661010* were the only DEGs not detected in other stages except for YT-f vs YT-p. Interestingly, the transcripts were also highly up-regulated in RT-f vs YT-p (Fig. 5), suggesting a potential involvement in transition from callus differentiation (f) to cotyledonary embryo formation (p) in YT. Important DEGs related to auxin pathway are resented in Fig. 5.

Together with endogenous auxin levels, it can be observed that in YT, IAA biosynthesis decreases in YT-y compared to YT-h, and then further increases in YT-f and YT-p stages, which can be attributed to the increased expression of the IAA biosynthesis genes TAA and YCC. Contrasting IAA accumulation and gene expression in RT, clearly suggest that IAA plays significant role in SE. Furthermore, the expression changes in IAA signaling genes confirm this proposition as detection of higher IAA changes the expression of downstream genes, thus suggesting that IAA could be a positive regulator of SE.

**Fig. 6:**
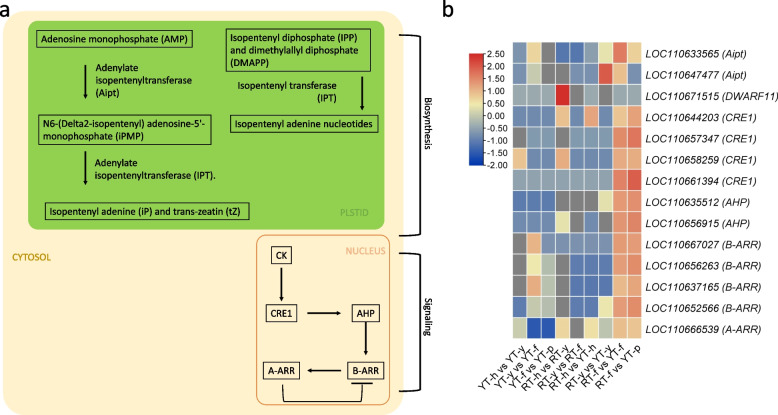
Cytokinin biosynthesis and signaling pathway. **a** representation of CK biosynthesis and signaling pathway genes and metabolites. **b** Heatmap representation of differentially expressed genes in CK biosynthesis and signaling pathway. Red: up-accumulation, Blue: down-accumulation, yellow: no variation of expression. SE: somatic embryogenesis, h: immature anther, y: anther-induced callus, f: callus differentiation stage, p: cotyledonary embryo

The biosynthesis of CKs starts with the production of isopentenyl diphosphate (IPP) and dimethylallyl diphosphate (DMAPP) through the mevalonic acid pathway or the non-mevalonic acid pathway [51]. The precursors IPP and DMAPP are then combined by an enzyme called isopentenyl transferase (IPT) to form isopentenyl adenine nucleotides, which are the primary forms of CKs in most plants. Our results did not show differential expression of the *IPT* gene (Fig. 6). However, there were two differentially up-regulated adenylate isopentenyl transferase (Aipt) transcripts (*LOC110633565* and *LOC110647477*) in YT-y vs YT-f. The expression of *LOC110633565* was higher in YT stages compared to those of RT. In addition to IPT and Aipt, other enzymes such as cytochrome P450 monooxygenase (also called Dwarf protein 11 or DWARF11; *LOC110671515*) were exclusive to RT (Fig. 6).

For the cytokinin signaling pathway (which is the part of main plant-hormone signaling pathway i.e., ko04075), eight DEGs annotated as CRE1 or histidine kinase 4 were identified. Only one DEG was found in the first two stages (Fig. 6). At this stage, the plant cells are still in a non-embryogenic state, and CK signalling is likely to be low. However, *LOC110644203, LOC110657347, LOC110658259,* and *LOC110661394* were up-regulated during RT-y vs YT-y and RT-y vs YT-p, which is consistent with the higher CK content in YT-y (Fig. 2e). Eleven DEGs annotated as histidine-containing phosphotransfer protein 1-like (AHP) were identified. Of these, *LOC110656915* and *LOC110635512* showed down-regulation from YT-h to YT-p but did not differentially express in RT. However, both AHPs were up-regulated in YT-f as compared to RT-f. Our results showed the differential expression of 38 B-ARRs. Among these B-ARRs, the transcription repressor KAN1 (*KANADI 1; LOC110652566, LOC110637165*), MYB family transcription factor (TF) (*PHL8-like*; *LOC110656263*), Protein ABERRANT TESTA SHAPE (*KANADI4; LOC110667027*) represented the most up-regulated DEGs in YT with an increasing expression trend. However, these showed decreasing expression trends in RT. Like AHPs, the B-ARRs were up-regulated in YT-f as compared to RT-f (Fig. 6b). Of the 10 A-ARRs, *ARR9* (*LOC110666539*) was down-regulated from YT-h to YT-p and up-regulated from RT-h to RT-y (Fig. 6). However, generally, its expression was higher in YT compared to RT. In summary, genes involved in CK signalling were globally up-regulated at callus differentiation stage (f) in YT compared to RT.

**Fig. 7:**
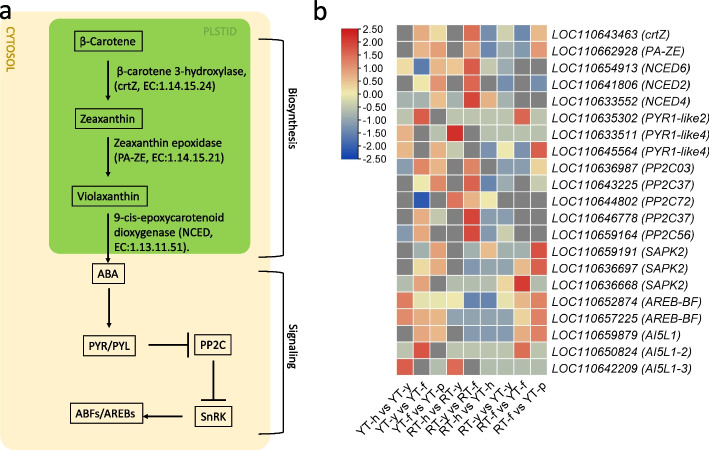
ABA biosynthesis and signaling pathway. **a** Representation of ABA biosynthesis and signaling pathway genes and metabolites. **b** Heatmap representation of differentially expressed genes in ABA biosynthesis and signalling pathway. Red: up-accumulation, Blue: down-accumulation, yellow: no variation of expression

In case of ABA biosynthetic pathway (which is the part of carotenoid biosynthesis pathway in plants i.e., ko00906), a beta-carotene 3-hydroxylase (*crtZ*: *LOC110643463*) was down-regulated during callus induction stages (YT-y and RT-y), and up-regulated during callus differentiation stage (YT-f and RT-f) (Fig. 7). Zeaxanthin epoxidase (*PA-ZE*: *LOC110662928*) was up-regulated during the YT-f and RT-f stages. For 9-*cis*-epoxycarotenoid dioxygenase (*NCED2*), three transcripts *(LOC110654913, LOC110641806,* and *LOC110633552*) were up-regulated during YT-f and RT-f stages, but their expression was higher in RT-f compared to YT-f, which is consistent with the ABA content in these SE stages (Fig. 2).

The ABA signaling pathway (which is the part of main plant-hormone signaling pathway i.e., ko04075) in plants involves a network of genes, including ABA receptors (PYR/PYL/RCARs), protein phosphatase 2Cs (PP2Cs), SNF1-related protein kinases 2 (SnRK2s), and ABA-responsive element binding factors (ABFs/AREBs) (Fig. 7). In the current study, 15 PYR/PYL genes were differentially expressed. The *LOC110635302, LOC110633511, LOC110645564* represented the most significant DEGs. Here, *LOC110635302* was the most up-regulated PYR/PYL in callus differentiation stages (f) of both genotypes (YT-y vs YT-f and RT-y vs YT-f). Whereas, of the 19 differentially expressed PP2Cs, five (*LOC110636987, LOC110643225, LOC110644802, LOC110646778,* and *LOC110659164*) genes were either down-regulated or weekly up-regulated in SE of YT. However, these were significantly up-regulated during RT-f. The SnRK2 transcripts (13 in total) demonstrated relatively lower differential expression. Though they had globally higher expression in YT compared to RT. Similarly, AREB TFs were significantly up-regulated in SE of YT as compared to RT (Fig. 7).

### Differential expression of genes enriched in MAPK signaling pathways

**Fig. 8:**
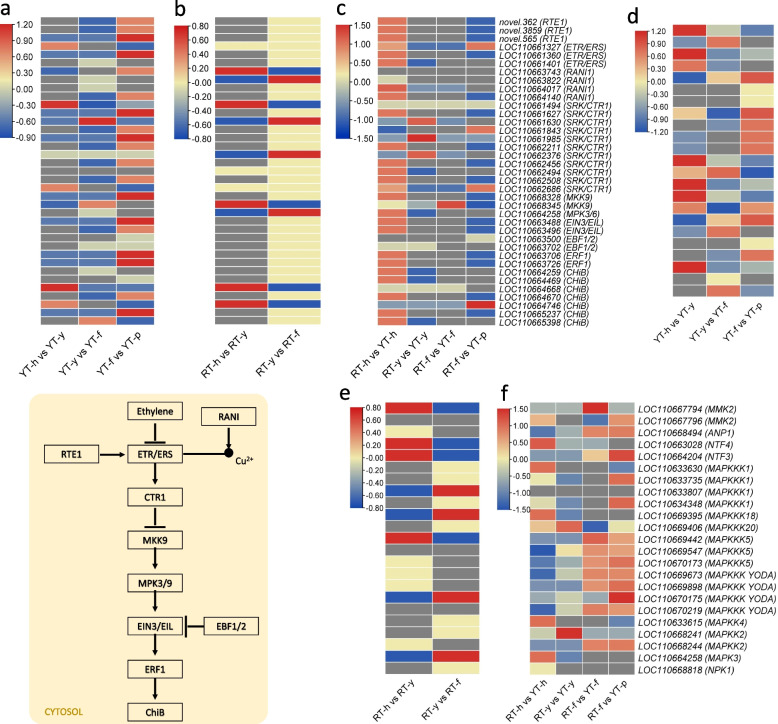
Differential expression of genes involved in ethylene signaling (**a**, **b**, and **c**) and MAPK signaling (**d**, **e**, and **f**). Red: up-accumulation, Blue: down-accumulation, yellow: no variation of expression. SE: somatic embryogenesis, h: immature anther, y: anther induced callus, f: callus differentiation stage, p: cotyledonary embryo

We found 630 DEGs associated with 40 KO terms that could be enriched in the MAPK signaling pathways. Of these, 625, 602, 604, and 620 DEGs were expressed in YT-h, YT-y, YT-f, and YT-p, respectively. On the other hand, 627, 605, and 590 DEGs were expressed in RT-h, RT-y, and RT-f, respectively. Of the 630 DEGs, 254 were commonly enriched in the MAPK signaling and plant-hormone signaling pathways. These DEGs were associated with 13 KO terms related to ETH, JA, and ABA signaling. Since we observed increased ETH contents in RT-f compared to no detection in YT-f, therefore, we specially looked at the genes related to ETH signaling. In total, there were 73 transcripts associated with 13 KO terms enriched in ETH signaling. Of these, 38 genes, associated with 10 KO terms had higher FPKM values in RT-f compared to YT-f. These genes include ethylene response regulator (*ERR*), serine/threonine-protein kinase (*SRK*), ethylene insensitive 3-like (*EIN3*), EIN3-binding F-box protein 1-like (*EBF1/2*), ethylene-response factor 1B-like, copper-transporting ATPase (*RANI1*), MAPK3, chitinase-like protein 1 (*CHiB1*), and protein REVERSION-TO-ETHYLENE SENSITITY 1 (*RTE1*) (Fig. 8a-c). The higher expression of these genes is consistent with the increased ETH content detected in RT-f compared to YT-f. Additionally, we observed that several ETH signaling genes showed decreasing expression in successive SE stages in YT but not in RT. These include SRKs (*STY8-like*, *LOC110662211*, *CTR1-like*, *LOC110662376*, and *HT1-like*, *LOC110662494*), *EIN3* (*LOC110663488*), *ERF 1B-like* (*LOC110663726*), *RANI1* (*HMA4-like*, *LOC110663743* and *PAA2*, *LOC110664140*), *MAPK3* (*LOC110664258*), and *CHiB2* (*LOC110664259* and *LOC110664469*) (Supplementary Table S3).

In addition to ETH signaling, we also looked for the changes in the expression of genes related to oxidative stress as this is known to induce SE [52]. In the MAPK signaling pathway, the oxidative stress is perceived as H_2_O_2_, which then activates a signaling cascade of MAPKs, SRKs (OXI1), and downstream genes. We observed the changes in the expression of 45 MAPKs including *MMK2, MAPK3, MAPKK* (2, 3, 4, 9, 10), *MAPKKK* (1, 5, 17, 18, 20, and YODA), *NTF3, 4, ANP1*, and *NPK1*. Interestingly, the expression of *ANP1* (*LOC110668494*), *MAPKK2* (*LOC110668244*), *MAPKK4* (*LOC110633615*), *MAPKKK YODA* (*LOC110669673, LOC110669898, LOC110670175, LOC110670219), MAPKKK5 (LOC110669442, LOC110669547,* and *LOC110670173*), *MMK2* (*LOC110667794* and *LOC110667796*), *NTF3* (*LOC110664204*), and *NTF4* (*LOC110663028*) increased from YT-h to YT-f but showed contrasting expressions in RT in the respective stages. However, these trends were not observed for all MAPKs. Particularly, we observed that *MAPK3*, *MAPKKK1*, *MAPKKK18*, and *NPK1* had higher expressions in YT than RT in stage h but for other stages (y, f, and p) their expressions were higher in RT than YT. These expression trends indicate that oxidative stress plays important role in SE in *H. brasiliensis,* particularly the genes showing increasing expression trends may be positive regulators of SE (Fig. 8d-f).

### Differential expression of somatic embryogenesis related genes

**Fig. 9:**
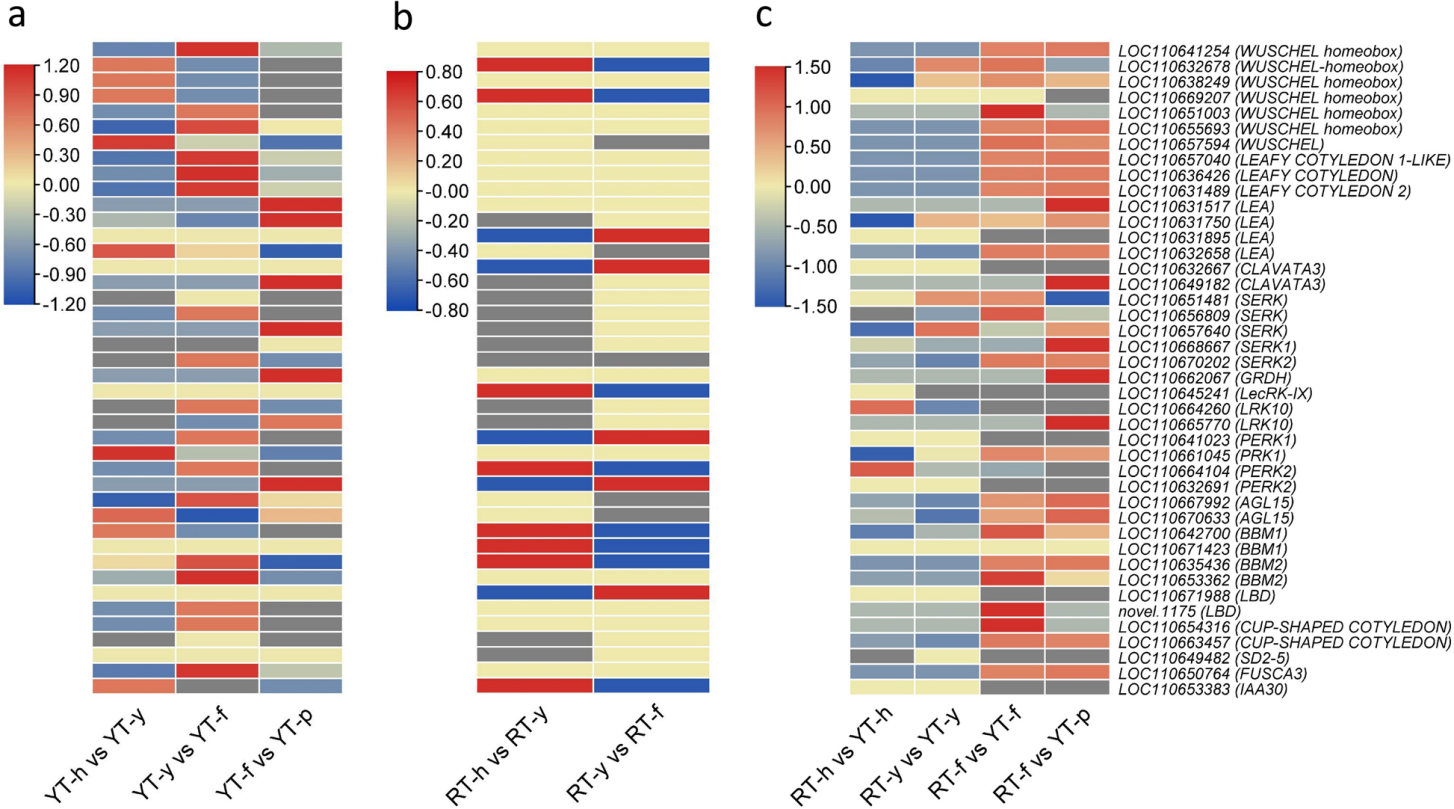
Expression profiling of SE related genes. **a** Gene expression analysis in YT and **b**) RT. **c** Comparative gene expression analysis during SE in both genotypes. Red: up-accumulation, Blue: down-accumulation, yellow: no variation of expression. SE: somatic embryogenesis; h: anther; y: embryogenic callus induction; f: callus differentiation for primary embryo; p: cotyledonary embryo

During SE, the WUS genes are expressed in embryogenic cells and are required to maintain their undifferentiated state. In comparative analysis of both genotypes, *WUS* gene expression was generally higher in later stages of YT compared to RT. The LEAFY COTYLEDON (*LEC*) TF has also been implicated in plant SE. Three transcripts of LEC genes (*LOC110657040, LOC110636426, LOC110631489*) were up-regulated during YT-y vs YT-f but were not differentially expressed in RT-y vs RT-f. This suggests a potentially important role for these genes in callus differentiation. Among the four differentially expressed *LEA* transcripts, *LOC110631517* was only up-regulated during YT-f vs YT-p. Similarly, *LOC110631895* was only up-regulated during RT-y vs RT-f (Fig. 9). Other than these genes, CLAVATA3 (*CLV3*) has been shown to play a role in regulating embryogenic competence and promoting the formation of somatic embryos. In the current study, *CLV3* (*LOC110632667*) was not detected in YT and up-regulated in RT-y vs RT-f. Another *CLV3* (*LOC110649182*) was highly up-regulated in RT-f vs YT-p (Fig. 9). Moreover, five *SERK* transcripts (*LOC110651481, LOC110656809, LOC110657640, LOC110668667, LOC110670202*) were generally down-regulated in SE in RT-y compared to RT-h. However, *LOC110670202* (*SERK2*) was significantly up-regulated during YT-y vs YT-f and down-regulated during same stage (RT-y vs RT-f) in RT.

The GRDH (glucose and ribitol dehydrogenase, *LOC110662067*) was up-regulated only during YT-f vs YT-p. Three transcripts (*LOC110645241, LOC110664260, LOC110665770*) of LERKs (L-type lectin-domain containing receptors also called SE receptor kinase 1) were globally up-regulated in RT compared to YT. On the other hand, AGL15 transcripts were significantly up-regulated in RT-f vs YT-f. Furthermore, four BBM transcripts (*LOC110642700, LOC110671423, LOC110635436, LOC110653362*) were differentially up-regulated during the early SE stages and then their expressions decreased. Interestingly, *BBM1* (*LOC110642700*) and *BBM2* (*LOC110635436*) were up-regulated in RT-f vs YT-f. Similarly, *LBD*, *CUP-SHAPED COTYLEDON*, and *FUSCA3* genes were globally significantly up-regulated in callus differentiation (YT-f) and somatic embryo (YT-p) stages. However, *SD2-5* and *IAA30* transcripts were up-regulated in RT-y and RT-f stages (Fig. 9). Overall, distinct gene expression profiles were observed for several SE-related genes in both genotypes, suggesting their potential roles in SE.

### Changes in H_2_O_2_ content

**Fig. 10:**
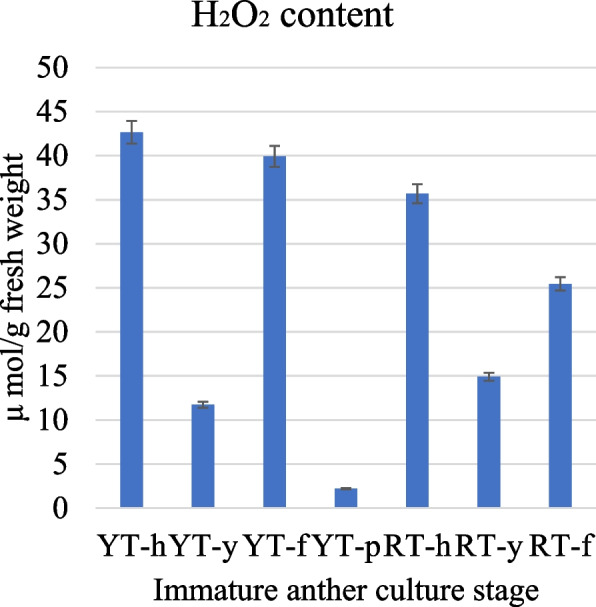
Relative H_2_O_2_ content in YT and HT genotypes during SE. Where h: anther; y: embryogenic callus induction; f: callus differentiation for primary embryo; p: cotyledonary embryo

Since the transcriptome expression suggested a possible role of oxidative stress in the differential SE of the two genotypes, we measured the H_2_O_2_ content. We observed that YT genotype had highest H_2_O_2_ content compared to RT. We observed that the content first decreased from “h” to “y” stages in both genotypes and then increased from “y” to “f” stages. However, the relative increase was higher in YT than in RT (Fig. 10). This validates the expression changes related to oxidative stress (Fig. 8d-f).

## Discussion

The most commonly used method in *Hevea* spp. propagation is grafting onto seedling rootstocks, but the plants developed by this method show interclonal variability due to the inherent genetic variability of the rootstocks [53]. SE is an important tool used for the propagation of plant species of economic and agronomic significance. It has been considered as a key technology to shape the future of Hevea due to its advantages such as regaining ontogenetic juvenility and production of transgenic lines [5]. To efficiently utilize SE in Hevea, it is essential to explore the differences in SE potential of the available genotypes. A previous transcriptome-based study reported the involvement of phytohormone signaling, phenylpropanoid biosynthesis, and starch and sucrose metabolism pathways in Hevea SE [20]. However, limited or no data is available on the differential accumulation of phytohormones in different stages involved in SE and the corresponding changes in the expression of biosynthetic genes. We used the UPLC-ESI–MS/MS method to determine the differential accumulation of phytohormones in different stages of somatic embryo formation from anthers of two genotypes with different embryogenesis capacity. The genotype YT exhibited a higher callus induction compared to RT (Fig. 1), such differences could be due to the genetic background as reported in cotton [54], ryegrass [55], and *Primula ssp.* [56] etc. Further, the absence of SE in RT, lead us to hypothesize that this could be due to differences in endogenous phytohormone content at different SE stages in the two genotypes. This hypothesis was based on the known role of phytohormones in differential SE in many plant species such as *Medicago truncatula* [57], cotton [54], and Korean pine [58]. In Hevea SE, a three decade old study investigated that the presence of auxins such as 3,4-dichlorophenoxyacetic acid or CKs can suppress SE [59], which is contrary to recent studies highlighting that IAA signaling genes are activated in SEs [20]. Thus, we further explored the expression changes in genes related to biosynthesis and signaling of phytohormones including IAA, CKs, ET, and ABA.

Among the phytohormones, auxins are key regulators of plant cell division and cell differentiation, and are therefore widely used for callus and SE induction. Thus, increased endogenous auxin levels are related to regulation of SE process [60]. Our observations that IAA levels increased from YT-y to YT-f are consistent with significantly increased expression of *TAA* and *YUC* in YT-f (Fig. 2c), which are genes involved in IAA biosynthesis. Among these, YUC genes are transcriptionally regulated by *BBM*, which in turn increases IAA biosynthesis and auxin response, as recently reported in Arabidopsis [61]. In addition to *BBM*, *YUCs* are also direct targets of *LECs*. When *LECs* were activated in Arabidopsis seedlings they induce SE and auxin biosynthesis [62]. Since these genes showed higher expressions in YT compared to RT, it is possible that SE in Hevea is *BBM* and *LEC* mediated. These genes induce higher auxin biosynthesis during SE, which in turn triggers a signaling cascade that affects the expression of several SE-related genes and initiate vegetative-to-embryogenic transition. This auxin induced transition is WUS-mediated [63]. Our observations that *WUS* genes had higher expressions in YT-h compared to RT-h are consistent with these reports as well as other reports in *M. truncatula* [64]*,* cotton [65], and coffee [66] (Fig. 9). These results indicate that auxin accumulates during SE progression in YT, but not in RT, and this accumulation is associated with changes in the expression of SE-related and auxin biosynthesis genes.

ETH plays a species-specific role in SE. Earlier studies on *Daucus carota* and* Coffea canephora* have shown that ETH reduces the SE potential [67, 68]. ETH overproducer Arabidopsis plants showed reduced free IAA levels [69]. This reduction was associated with the increased expression of *AUX1 influx carriers* that are responsible for increased auxin transport [70]. Our observations that ETH was not detected in YT-f, in contrast to relatively higher levels in RT-f, and the contrasting IAA levels (Fig. 2) in the two genotypes are consistent with the reports on *D. carota* and *C. canephora.* These observations suggest that ETH could be a possible cause of the absence of SE in the RT genotype. The relatively higher FPKM value of three *AUX1/LAX* genes and 38 ETH signaling related genes in RT-f compared to YT-f are also consistent with these findings (Supplementary Table S3) [70]. Thus, our results indicate that the genotype with no SE potential shows higher ETH content at the “f” stage compared to no content in YT at the corresponding stage. This higher ETH triggers downstream signaling genes. Hence, we propose that the absence of ETH in YT-f could be another possible cause of SE, as observed in black spruce, carrot, and coffee [67, 68, 71].

Studies have shown that ABA is associated with embryo development and maturation [72]. We also observed an increase in relative ABA levels from “y” to “f” stages of both genotypes (Fig. 2). In cotton, an appropriate concentration of ABA could promote SE [22]. Endogenous ABA levels are under the coordinated control of multiple factors, including its biosynthesis, transport, and stresses [73]. The increase in the expression of cZ from y to f stages together with higher expression of *PA-ZE* and *NCED2* in YT-f and RT-f is consistent with the observed ABA levels (Figs. 2 and 7). *PA-ZE* converts zeaxanthin to violaxanthin, which is a key reaction in ABA biosynthesis [74], whereas NCED catalyzes the steps that convert neoxanthin to ABA [75]. The ABA accumulation triggers storage proteins and LEAs, suggesting that LEAs are components of ABA-inducible systems [76]. Since both genotypes had specific LEA transcripts that were differentially expressed (Fig. 9), it is premature to define and specify how ABA plays a role in SE in Hevea. However, the accumulation trends and expression changes clearly indicate that ABA plays a positive role in SE. Experiments specially targeting ABA in SE, and characterizing the key biosynthesis and signaling genes would significantly improve our understanding about SE in Heavea.

Auxins and CKs are known for their key roles as regulators of both cell division and differentiation in plants [77]. The significant increase in tZ levels in YT-y (Fig. 2) are consistent with earlier reports that CK is regulator of the shoot apical meristem [78]. This higher tZ content in YT could be due to the up-regulation of *Aipt* in YT compared to RT because this gene converts adenosine monophosphate into N6-(delta2-isopentenyl) adenosine-5'-monophosphate, which can be further phosphorylated and dephosphorylated to form various forms of CKs i.e., tZ [79]. Moreover, the differences in CK levels may also be due to the expression of different CK synthesis genes in YT and HT (Fig. 6) [80]. Nevertheless, the reducing levels of cZ and presence of higher quantities of tZ at the YT-y stage indicate their important role in SE. The higher CK content in earlier stages of SE observed in our results indicate that CK is required for cell proliferation, probably to increase mass [81]. Since from “y” to “f”, the callus starts dedifferentiation, and the fact that CKs, if produced constantly, they can produce irregular cell division. Hence the observed reduced content of CKs in latter SE stages is understandable [51]. The opposite auxin and CK accumulation trends suggest that CKs also regulate local auxin metabolism and modulate auxin pools in Hevea, as in other plants [82]. However, this needs further detailed exploration by modulating exogenous concentrations of the two hormones. The levels of CKs are perceived by CK signaling genes such as AHPs, which act as CK receptors [83], and the reduction in AHPs expression at YT stages is consistent with the decreasing trends in CK accumulation. After sensing, the CK response is mediated by A-ARRs (positive regulators) and B-ARRs (negative regulators) [84]. Thus the contrasting expression trends of genes annotated as A-ARRs and B-ARRs during SE are consistent with earlier reports [85]. Taken together, the accumulation trends of CKs and the expressions of biosynthesis and signalling genes indicate that CKs together with auxin, play significant role in SE. Particularly, our results that CKs and auxin had opposite accumulation trends are consistent with their antagonistic roles in plant development [51]. Since our results provide a broader understanding based on the expressed transcripts (genes) and accumulation trends, therefore, to underpin the specific role(s) of each of these phytohormones gene characterization studies would be needed.

Finally, in addition to stress stimuli, reactive oxygen species (ROS) are thought to be ubiquitous in normal cells and act as cellular messengers that can induce gene expression leading to SE in plants [52, 86]. In particular, hydrogen peroxide (H_2_O_2_) has been implicated in SE in recent years [87]. In the MAPK signaling pathway, H_2_O_2_ is perceived as an intracellular messenger and triggers a signal cascade. The up-regulation of *MMK2*, *MAPK3*, *MAPKKs*, *MAPKKKs*, *NTF3*, *ANP1*, and *NPK1* from YT-h to YT-f indicates that H_2_O_2_ sensing is increased at this stage, inducing the expression of these signaling genes (Fig. 8). This was further confirmed by the observation that changes in H_2_O_2_ content were consistent to these expression changes (Fig. 9). Thus, our results provide preliminary understanding that an increased oxidative stress can also be a possible reason for SE in YT. Previous studies have shown a positive correlation between SE and H_2_O_2_ in *Lycium barbarum* [88]. Similarly, H_2_O_2_ has been reported to play a key role in conversion of embryogenic callus into somatic embryos [89]. The fact that these genes were significantly up-regulated in YT-f suggests that H_2_O_2_ may act as a messenger activating the expression of genes and the biosynthesis of proteins required to regulate the morphogenetic pathways leading to SE [90, 91].

## Conclusions

We conducted a comparative transcriptome and phytohormone analysis using YT and RT Hevea genotypes with contrasting SE potential. The results indicate that YT has a higher callus induction rate (%) compared to RT and leads to SE, which is absent in RT. The UPLC-ESI–MS/MS system detected seven phytohormones of which IAA, CKs (tZ and cZ), ABA, and ETH showed differential accumulation trends. Phytohormone biosynthesis and signal transduction gene expression confirmed the UPLC-ESI–MS/MS results. Based on the two technologies applied, we conclude that the higher IAA content in YT compared to RT was associated with the higher expression of IAA biosynthesis, hence it could be a positive regulator of SE. Similarly, the detection of ETH in RT but not in YT at “f” stage and the corresponding gene expression changes may be related to the SE potential of the two genotypes. Finally, the accumulation trends of the CKs and the expressions of biosynthesis and signalling genes indicate that CKs play an important role in SE; most probably as a negative regulator. Our results also suggest the possible role of H_2_O_2_ in SE in Hevea.

### Supplementary Information

**Additional file 1: Supplementary Table 1.** Relative phytohormone based on UPLC-ESI-MS/MS in YT and HT genotypes at four stages of somatic embryogenesis of *Hevea* genotypes YT and RT.

**Additional file 2: Supplementary Table 2.** Statistics of transcriptome sequencing of 21 libraries belonging to four stages of somatic embryogenesis of *Hevea* genotypes YT and RT.

**Additional file 3: Supplementary Table 3.** Expression of genes enriched in MAPK signaling pathway in four stages of somatic embryogenesis of *Hevea* genotypes YT and RT.

**Additional file 4: Supplementary Figure 1.** Details of metabolites identified related to IAA biosynthesis, storage and degradation.

**Additional file 5: Supplementary Figure 2.** Metabolites related to CK biosynthesis and degradation.

**Additional file 6: Supplementary Figure 3.** Classification of KEGG pathways to which the DEGs in RT-h vs RT-y and RT-y vs RT-f were enriched.

**Additional file 7: Supplementary Figure 4.** Classification of KEGG pathways to which the DEGs in YT-h vs YT-y, RT-y vs RT-f, and YT-f vs YT-p were enriched.

## Data Availability

The raw sequencing data are available at NCBI SRA under the project number: PRJNA963065 (https://www.ncbi.nlm.nih.gov/bioproject/963065).
