# Supplementary material for: Comparative transcriptome analysis of high- and low-embryogenic Hevea brasiliensis genotypes reveals involvement of phytohormones in somatic embryogenesis
Source: BMC Plant Biol. 2023 Oct 13;23:489. doi: 10.1186/s12870-023-04432-3 (PMC10571474; doi:10.1186/s12870-023-04432-3)
Supplement: Supplementary file 4 — Additional file 4: Supplementary Figure 1. Details of metabolites identified related to IAA biosynthesis, storage and degradation. [file 12870_2023_4432_MOESM4_ESM.pptx]

## Slide 1
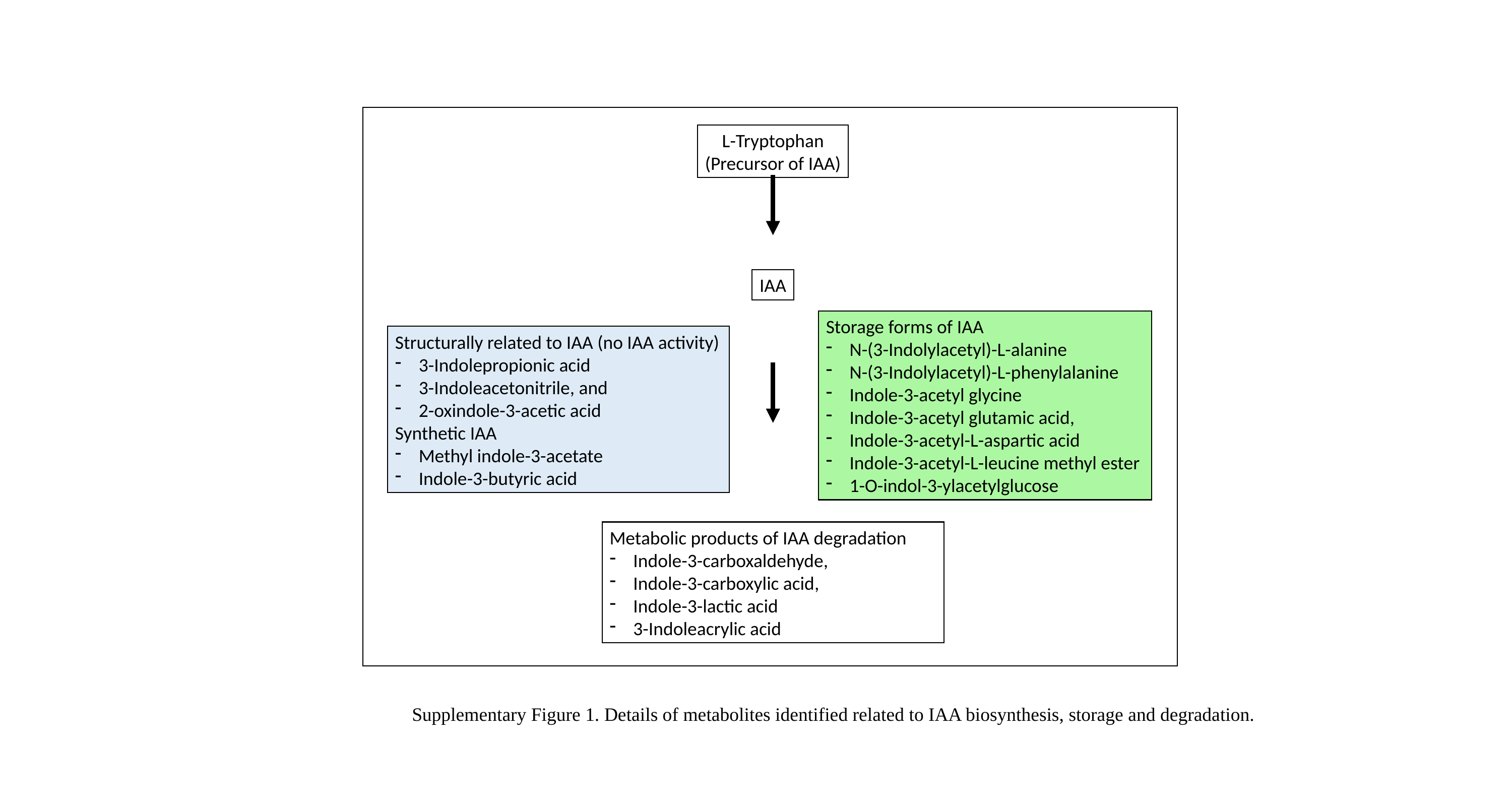

L-Tryptophan
(Precursor of IAA)
IAA
Storage forms of IAA
N-(3-Indolylacetyl)-L-alanine
N-(3-Indolylacetyl)-L-phenylalanine
Indole-3-acetyl glycine
Indole-3-acetyl glutamic acid,
Indole-3-acetyl-L-aspartic acid
Indole-3-acetyl-L-leucine methyl ester
1-O-indol-3-ylacetylglucose
Structurally related to IAA (no IAA activity)
3-Indolepropionic acid
3-Indoleacetonitrile, and
2-oxindole-3-acetic acid
Synthetic IAA
Methyl indole-3-acetate
Indole-3-butyric acid
Metabolic products of IAA degradation
Indole-3-carboxaldehyde,
Indole-3-carboxylic acid,
Indole-3-lactic acid
3-Indoleacrylic acid
Supplementary Figure 1. Details of metabolites identified related to IAA biosynthesis, storage and degradation.
