# Supplementary material for: Comparative transcriptome analysis of high- and low-embryogenic Hevea brasiliensis genotypes reveals involvement of phytohormones in somatic embryogenesis
Source: BMC Plant Biol. 2023 Oct 13;23:489. doi: 10.1186/s12870-023-04432-3 (PMC10571474; doi:10.1186/s12870-023-04432-3)
Supplement: Supplementary file 5 — Additional file 5: Supplementary Figure 2. Metabolites related to CK biosynthesis and degradation. [file 12870_2023_4432_MOESM5_ESM.pptx]

## Slide 1
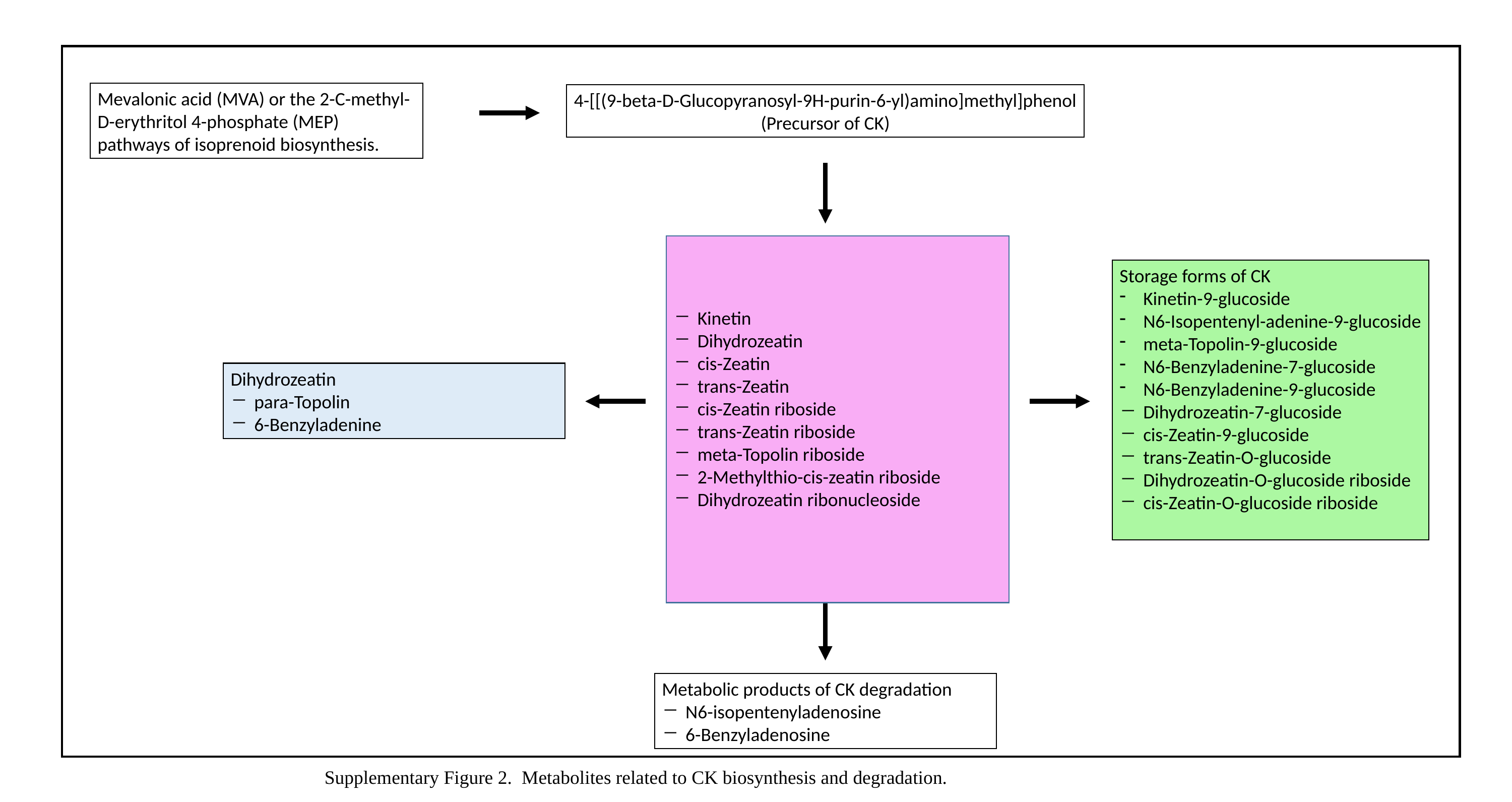

Mevalonic acid (MVA) or the 2-C-methyl-D-erythritol 4-phosphate (MEP) pathways of isoprenoid biosynthesis.
4-[[(9-beta-D-Glucopyranosyl-9H-purin-6-yl)amino]methyl]phenol
(Precursor of CK)
Kinetin
Dihydrozeatin
cis-Zeatin
trans-Zeatin
cis-Zeatin riboside
trans-Zeatin riboside
meta-Topolin riboside
2-Methylthio-cis-zeatin riboside
Dihydrozeatin ribonucleoside
Storage forms of CK
Kinetin-9-glucoside
N6-Isopentenyl-adenine-9-glucoside
meta-Topolin-9-glucoside
N6-Benzyladenine-7-glucoside
N6-Benzyladenine-9-glucoside
Dihydrozeatin-7-glucoside
cis-Zeatin-9-glucoside
trans-Zeatin-O-glucoside
Dihydrozeatin-O-glucoside riboside
cis-Zeatin-O-glucoside riboside
Dihydrozeatin
para-Topolin
6-Benzyladenine
Metabolic products of CK degradation
N6-isopentenyladenosine
6-Benzyladenosine
Supplementary Figure 2. Metabolites related to CK biosynthesis and degradation.
