# Supplementary material for: Comparative transcriptome analysis of high- and low-embryogenic Hevea brasiliensis genotypes reveals involvement of phytohormones in somatic embryogenesis
Source: BMC Plant Biol. 2023 Oct 13;23:489. doi: 10.1186/s12870-023-04432-3 (PMC10571474; doi:10.1186/s12870-023-04432-3)
Supplement: Supplementary file 6 — Additional file 6: Supplementary Figure 3. Classification of KEGG pathways to which the DEGs in RT-h vs RT-y and RT-y vs RT-f were enriched. [file 12870_2023_4432_MOESM6_ESM.pptx]

## Slide 1
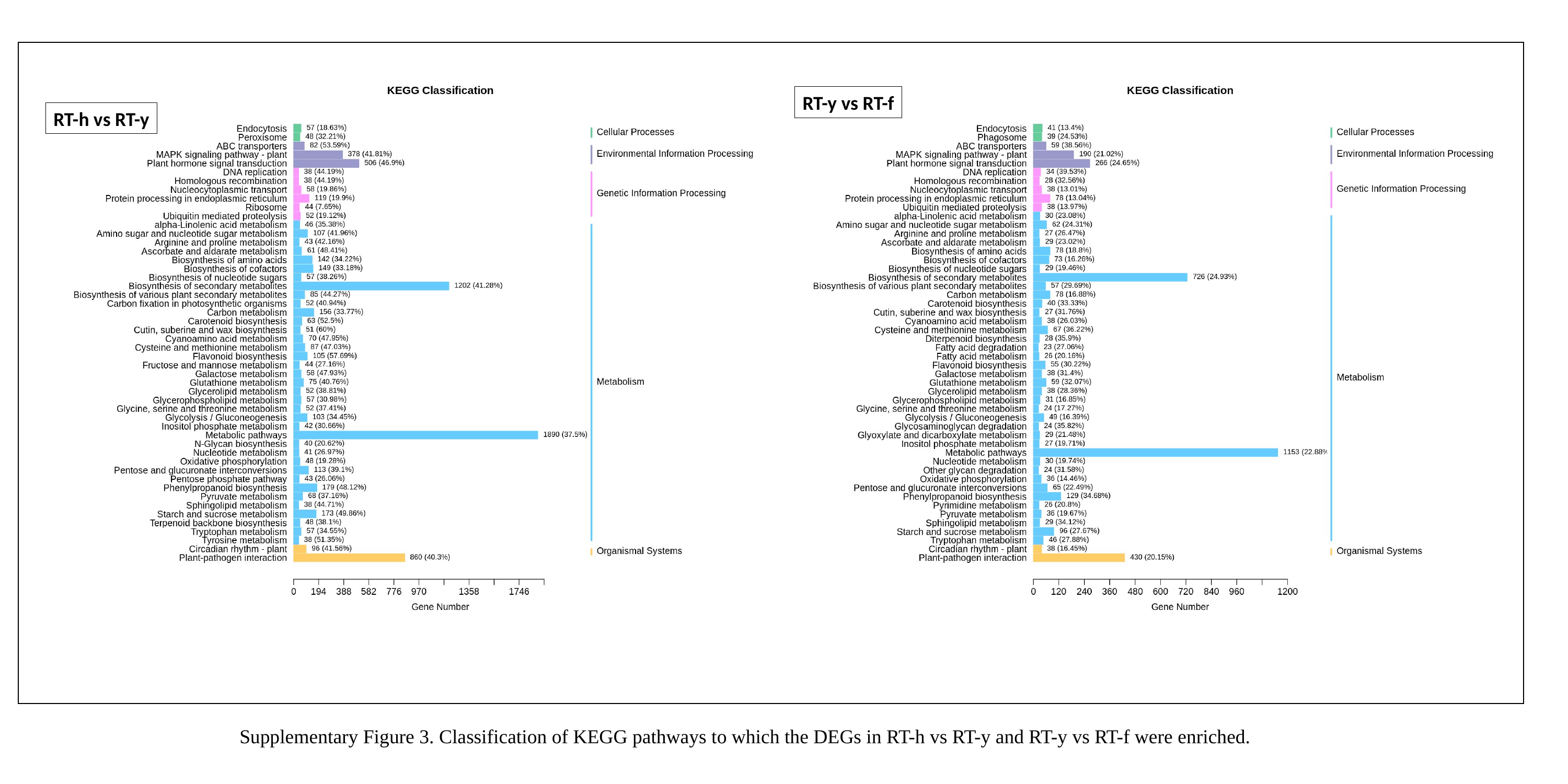

RT-y vs RT-f
RT-h vs RT-y
Supplementary Figure 3. Classification of KEGG pathways to which the DEGs in RT-h vs RT-y and RT-y vs RT-f were enriched.
