# Supplementary material for: Comparative transcriptome analysis of high- and low-embryogenic Hevea brasiliensis genotypes reveals involvement of phytohormones in somatic embryogenesis
Source: BMC Plant Biol. 2023 Oct 13;23:489. doi: 10.1186/s12870-023-04432-3 (PMC10571474; doi:10.1186/s12870-023-04432-3)
Supplement: Supplementary file 7 — Additional file 7: Supplementary Figure 4. Classification of KEGG pathways to which the DEGs in YT-h vs YT-y, RT-y vs RT-f, and YT-f vs YT-p were enriched. [file 12870_2023_4432_MOESM7_ESM.pptx]

## Slide 1
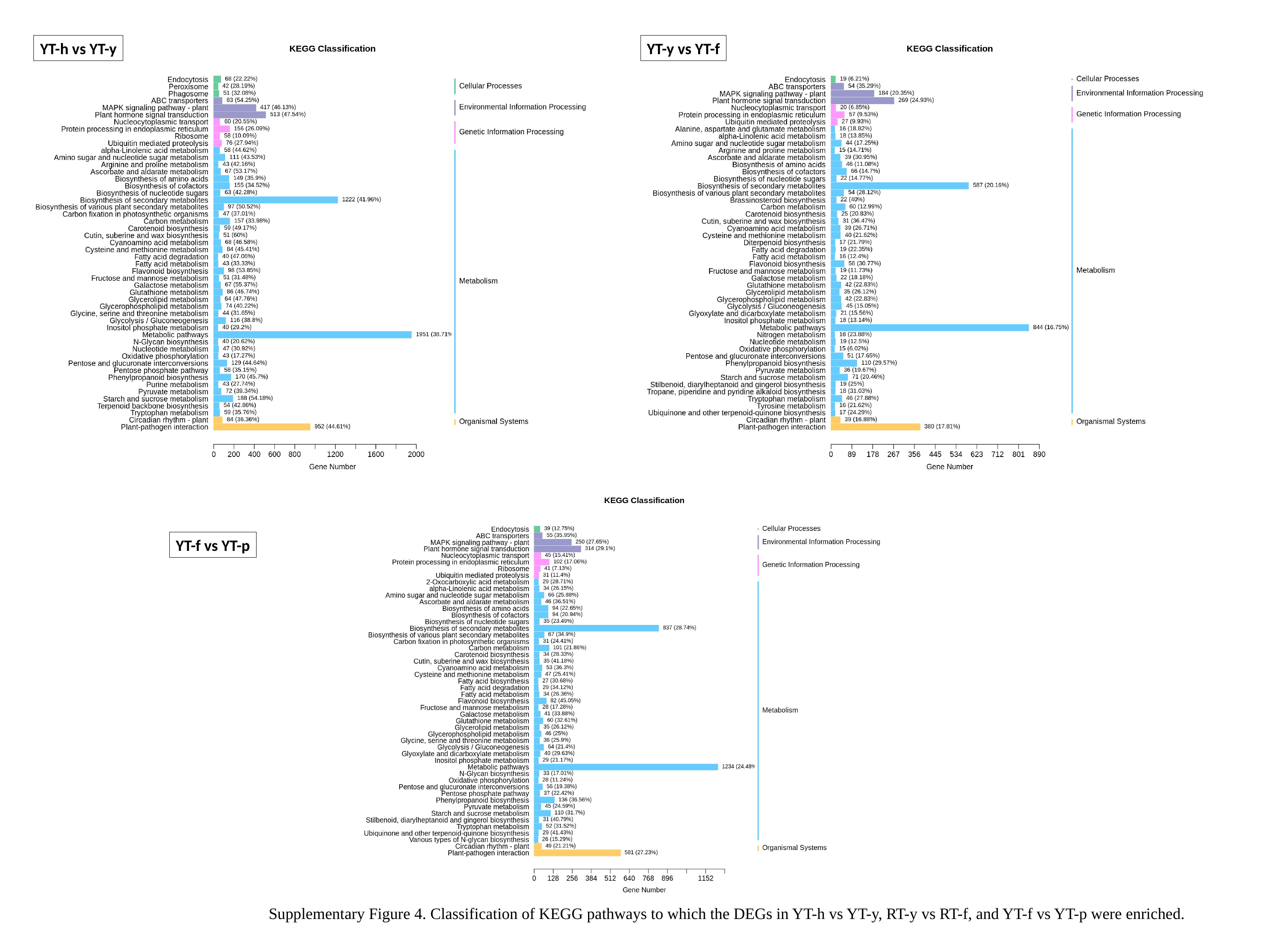

YT-h vs YT-y
YT-y vs YT-f
YT-f vs YT-p
Supplementary Figure 4. Classification of KEGG pathways to which the DEGs in YT-h vs YT-y, RT-y vs RT-f, and YT-f vs YT-p were enriched.
